# Sialyllactose suppresses angiogenesis by inhibiting VEGFR-2 activation, and tumor progression

**DOI:** 10.18632/oncotarget.16192

**Published:** 2017-03-14

**Authors:** Tae-Wook Chung, Eun-Young Kim, Seok-Jo Kim, Hee-Jung Choi, Se Bok Jang, Keuk-Jun Kim, Sun-Hyung Ha, Fukushi Abekura, Choong-Hwan Kwak, Cheorl-Ho Kim, Ki-Tae Ha

**Affiliations:** ^1^ School of Korean Medicine, Healthy Aging Korean Medical Research Center, Pusan National University, Yangsan, Gyeongsangnam-do 50612, Republic of Korea; ^2^ Graduate Training Program of Korean Medicine for Healthy-Aging, Pusan National University, Yangsan, Gyeongsangnam-do 50612, Republic of Korea; ^3^ Department of Biological Science, Sungkyunkwan University, Suwon, Kyunggi-do 16419, Republic of Korea; ^4^ Department of Molecular Biology, College of Natural Sciences, Pusan National University, Geumjeong-gu, Busan 46241, Republic of Korea; ^5^ Department of Clinical Pathology, TaeKyeung University, Gyeongsan 38547, Republic of Korea

**Keywords:** sialyllactose, milk, oligosaccharides, angiogenesis, VEGF receptor-2

## Abstract

The oligosaccharides in human milk have various biological functions. However, the molecular mechanism(s) underlying the anti-angiogenic action of sialylated human milk oligosaccharides (HMOs) are still unclear. Here, we show that siallylactose (SL) found in human milk can inhibit the activation of vascular endothelial growth factor (VEGF)-mediated VEGF receptor-2 (VEGFR-2) by binding to its VEGF binding site (second and third IgG-like domains), thus blocking downstream signal activation. SL also inhibits growth of VEGF-stimulated endothelial cells. In endothelial cells treated with VEGF, SL diminished tube formation, migration, and the arrangement of actin filament. In addition, SL clearly suppressed VEGF-induced neovascularization in an *in vivo* Matrigel plug assay. Notably, SL prevented the growth of tumor cells, and angiogenesis on tumor tissues in *in vivo* mice models allotransplanted with Lewis lung carcinoma, melanoma, and colon carcinoma cells. Taken together, we have demonstrated that the sialylated milk oligosaccharide sialyllactose functions as an inhibitor of angiogenesis through suppression of VEGF-mediated VEGFR-2 activation in endothelial cells, Accordingly, it could be a novel candidate for the development of anti-angiogenic drugs without any side effects.

## INTRODUCTION

Human milk is composed of many bioactive ingredients, including milk oligosaccharides [1, 2]. It has been suggested that human milk oligosaccharides (HMOs) have diverse biological functions. They can function as including prebiotics, anti-microbials, modulators of intestinal epithelial cell, and regulators of the immune system [3, 4]. HMOs form a complex group of oligosaccharides composed of more than a hundred different glycans. They are constructed by five basic monosaccharide elements, including glucose (Glc), galactose (Gal), *N*-acetylglucosamine (GlcNAc), fucose (Fuc), and sialic acid (Sia) [4]. HMOs are contained of lactose on the reducing end, a polylactosamine core, and often fucose or sialic acid at the nonreducing terminus [5, 6]. Among them, sialylated HMOs have unique biological functions, such as inhibition of cell-cell interaction and pathogen-host interaction, because of their negative charge and hydrophilic nature [3, 7, 8]. Cell surfaced glycans, especially sialic acid-containing oligosaccharides, play critical roles in the progression of tumorigenesis, including neoplastic transformation, survival, tumor-induced immune modulation, and metastasis [9]. Acetamido GM3 (a-GM3 including lactose moiety with sialic acid) which forms the core glycan component of the gangliosides, interacts with human galectin-3 associated with diseases including cancer, inflammation, and angiogenesis [10].

Several studies have demonstrated that HMOs have a suppressive effect on carcinogenesis and tumor growth [11, 12]. Furthermore, Rudolff *et al*. [13] demonstrated that sialylated HMOs have anti-angiogenic activity. However, the exact composition of oligosaccharides and underlying molecular mechanism responsible for the anti-angiogenic action of sialylated HMOs are still unclear.

In this study, we showed sialyllactose, a basic unit of sialiylated HMOs, has a suppressive effect on the phosphorylation of VEGFR-2 in endothelial cells through direct binding to the extracellular domain of the receptor. Sialyllactose inhibits angiogenesis *in vitro* and *in vivo*, and consequently reduces the growth of allograft tumors. These findings indicate that sialyllactose can be a novel anti-angiogenic drugs candidate.

## RESULTS

### Sialyllactose suppresses VEGFR-2 phosphorylation in HUVECs

**Figure 1:**
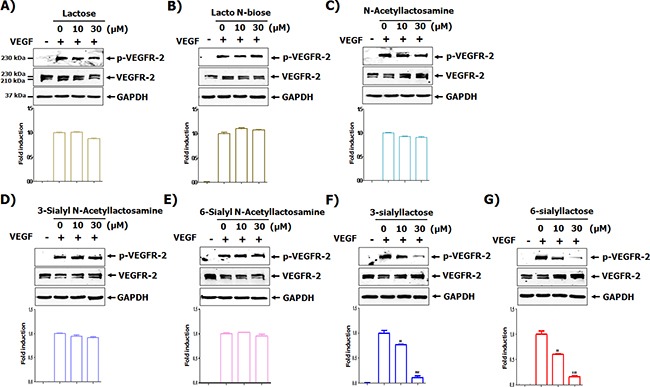
Screening of milk sialic oligosaccharides for their ability to inhibit VEGFR-2 activation **(A-G)** HUVECs were starved in EBM-2 containing 1 % FBS for 6 h. The cells were pretreated with indicated concentrations of each oligosaccharide for 1 h and stimulated with VEGF (50 ng/mL) for 15 min. The phosphorylation of VEGFR-2 was determined by western blot analysis. Densitometric analysis was performed and the relative value of p-VEGFR-2/ VEGFR-2/GAPDH is shown as fold change compared to the positive control. ** *p*<0.01 and *** *p*<0.001 compared to positive control (2^nd^ lane).

Although more than 150 oligosaccharides exist in HMOs, there are several structural core units, such as lactose, lacto *N*-biose, *N*-acetyllactosamine, 3′-sialyl-*N*-lactosamine (3SNL), 6′-sialyl-*N*-lactosamine (6SNL), 3′-sialyllactose (3SL), and 6′-sialyllactose (6SL) (Supplementary Figure 1) [2, 4]. As shown in (Supplementary Figure 1), basic HMOs did not affect on the cytotoxicity of HUVECs. However, among the basic HMOs, 3SL and 6SL have a dose-dependent inhibitory effect on the phosphorylation of VEGFR-2 in VEGF-stimulated HUVECs (Figure 1F and 1G). As shown in (Figure 1), 3SL and 6SL, composed of sialic acid, galactose, and glucose, both inhibited VEGF-induced VEGFR-2 activation. Accordingly, we used 3SL in subsequent experiments.

### Sialyllactose inhibits VEGF-induced growth, tube formation, migration and formation of actin stress fibers in HUVECs

**Figure 2:**
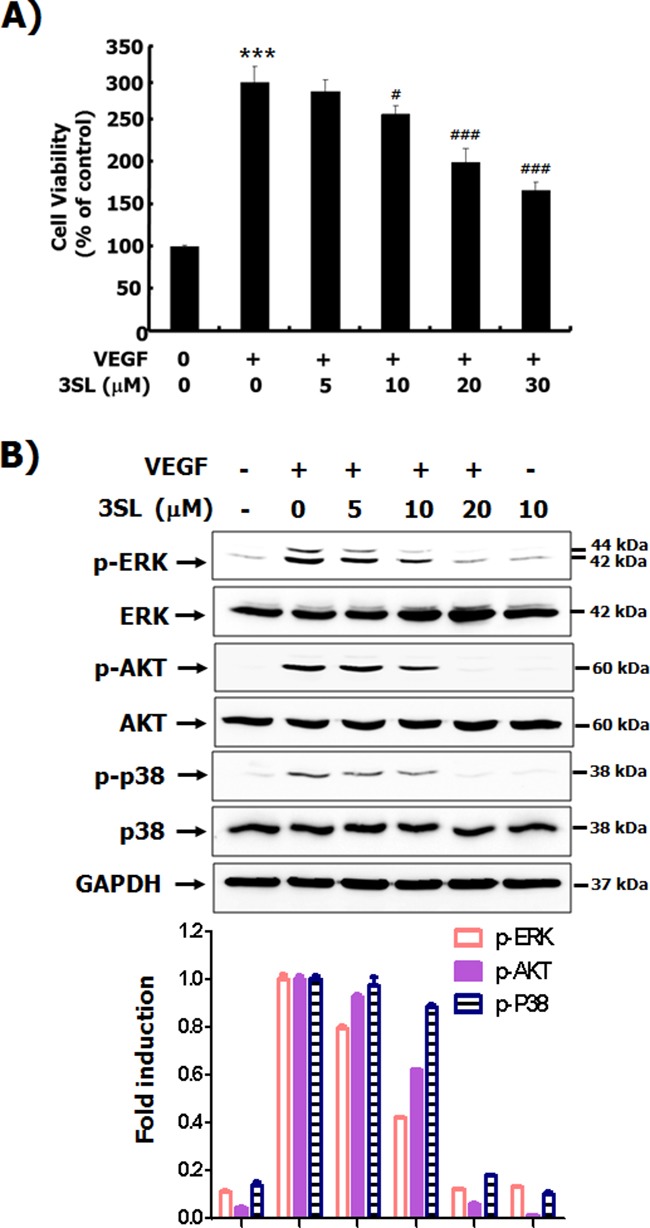
Effects of sialyllactose on the viability of HUVECs and activation of signal pathways stimulated by VEGF **(A)** HUVECs were treated with the indicated concentrations of sialyllactose in the presence or absence of VEGF (50 ng/mL) for 72 h. The effect of sialyllactose on the viability of VEGF-treated HUVECs was measured by MTT assay and are presented as means ± SD. *** *p*<0.001 compared to control (1^st^ lane), # *p*<0.05 and ### *p*<0.001 compared to the positive control (2^nd^ lane). **(B)** The HUVECs were starved in EBM-2 containing 1 % FBS for 6 h. The cells were pretreated with indicated concentrations of each oligosaccharide for 1 h and stimulated with VEGF (50 ng/mL) for 15 min. The phosphorylation of ERK, AKT, and p38 was analyzed by western blot analysis. Densitometric analysis was performed and the relative value of p-ERK/ERK/GAPDH, p-AKT/AKT/GAPDH, and p-p38/p38/GAPDH is shown as fold change relative to the positive control.

**Figure 3:**
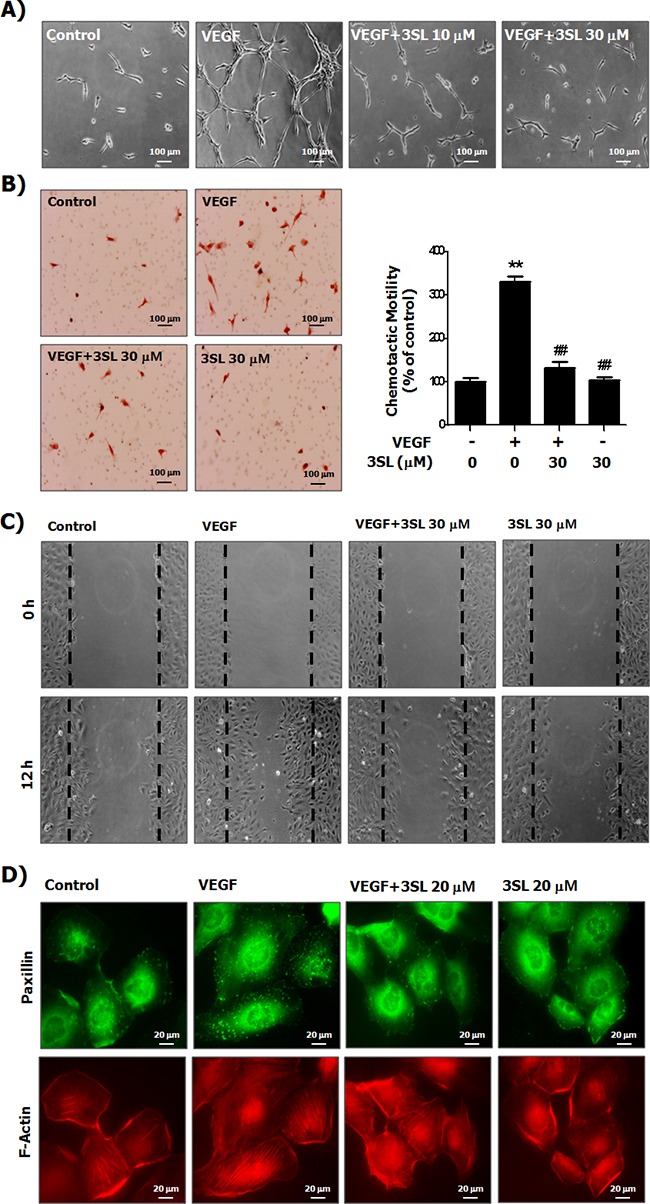
Suppressive effects of sialyllactose on tube formation, migration and formation of the actin stress fibers of HUVECs **(A)** HUVECs cultured on matrigel coated plates were treated with the indicated concentrations of sialyllactose and stimulated with VEGF (50 ng/mL) for 15 h. Morphological changes in HUVECs were observed by microscopy and representative images are shown. **(B)** HUVECs were seeded in the upper part of the Boyden chamber and treated with the indicated concentrations of sialyllactose in upper chamber. VEGF (50 ng/mL) was added to the lower compartment of the chamber. The chambers were incubated at 37°C for 24 h in a 5% CO_2_ atmosphere. The migrated cells stained with hematoxylin and eosin were imaged and counted. The numbers of migrated HUVECs are shown as the means ± SD. ** *p*<0.01 compared to the control (1^st^ lane) and ## *p*<0.01 compared to the positive control (2^nd^ lane). **(C)** The HUVECs were confluently grown. Monolayers of HUVECs were wounded. The cells were treated with the indicated concentrations of sialyllactose and stimulated with VEGF (50 ng/mL) for 12 h. **(D)** The formation of HUVEC actin stress fibers was analyzed by staining paxillin and F-actin using a specific antibody and phalloidin, respectively.

Although sialyllactose did not induce cytotoxicity of HUVECs (Supplementary Figure 1F and 1G), 3SL inhibited VEGF-stimulated growth of HUVECs in a dose-dependent manner (Figure 2A and Supplementary Figure 2). In addition to its inhibitory effect on the phosphorylation of VEGFR-2, 3SL suppressed the activation of signaling pathways downstream of VEGFR-2, including ERK, Akt, and p38. These pathways are related to proliferation, migration, formation of actin filaments and tube formation of VEGF-induced HUVECs (Figure 2B). The capillary-like tube formation of HUVECs induced by VEGF was also reduced by 3SL treatment (Figure 3A). Furthermore, VEGF-stimulated migration of HUVECs was remarkably decreased with 3SL treatment (Figure 3B). 3SL also inhibited VEGF-induced migration of HUVECs, as determined by wound healing assay (Figure 3C). Moreover, 3SL effectively suppressed VEGF-induced marked formation of actin filaments in HUVECs, and inhibited VEGF-stimulated the recruitment of paxillin to filamentous arrays, indicating contribution to cell migration (Figure 3D).

### Sialyllactose directly binds to extracellular domain (ExD) of VEGFR-2

**Figure 4:**
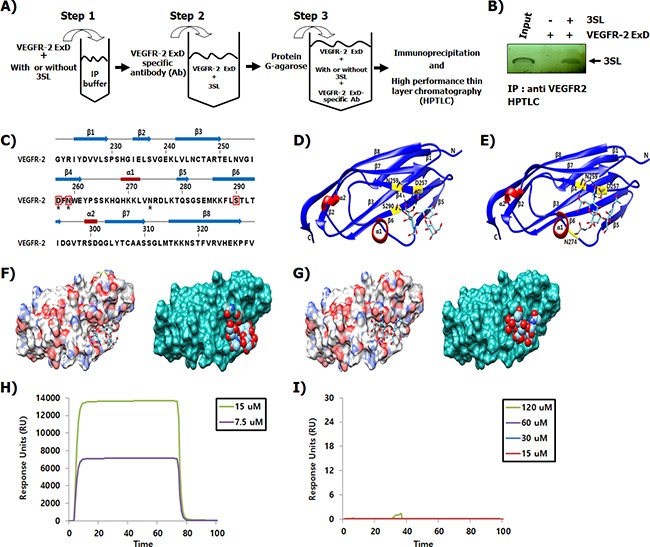
Binding of 3SL to the ExD of VEGFR-2 **(A)** Schematic presentation of the experimental procedure demonstrating the binding between sialyllactose and the extracellular domain of VEGFR-2. **(B)** Sialyllactose bound to the ExD of VEGFR-2 and the complex was immunoprecipitated using a VEGFR-2 specific antibody. After washing, sialyllactose was released and analyzed by HPTLC. Lane 1 indicates that the HPTLC loaded with SL as a loading control is developed with a resorcinol-hydrochloric acid reagent. **(C)** Sequence alignment and the secondary structure of the VEGFR-2 third IgG-like domain are shown. Secondary structure elements are indicated with the arrow (β-sheets) and rectangle (α-helix). The linker loops are shown as gray lines. Every ten residues are indicated by a bar. The interaction residues of 3SL and sialyl *N*-actetyllactosamine are indicated by red boxes and black stars, respectively. **(D-E)** Ribbon representations of the VEGFR-2 structure with 3SL or sialyl *N*-actetyllactosamine are shown. **(F)** Surface diagrams of VEGFR-2 with 3SL bound at the pocket (left: stick model, right: space filling shaped). Carbon atoms in gray, oxygen atoms in red, nitrogen atoms in blue, and sulfur atom in gold are shown. **(G)** Surface representation of VEGFR-2 with sialyl *N*-actetyllactosamine is shown. The interaction of **(H)** 3SL and **(I)** 3-sialyl *N*-actetyllactosamine with the 2-3 Ig-like domain of VEGFR-2 was measured using the Biocore assay.

By immunoprecipitation and high performance thin layer chromatography (HPTLC), we examined whether the inhibitory effect of 3SL on VEGFR-2 phosphorylation is due to direct interaction with the ExD of VEGFR-2 (Figure 4A). The results clearly demonstrated that 3SL interacted with the ExD of VEGFR-2 (Figure 4B). Next, we modeled the interaction of 3SL with the ExD of VEGFR-2 (PDB ID: 3S35), following a protein-small molecule docking method (Figures 4C-4G). Notably, 3SL bound to the purified second and third IgG-like domains of VEGFR-2, as validated by Biacore assay, but 3SNL did not (Figure 4H and 4I).

### Sialyllactose inhibits angiogenesis and tumor growth *in vivo*

**Figure 5:**
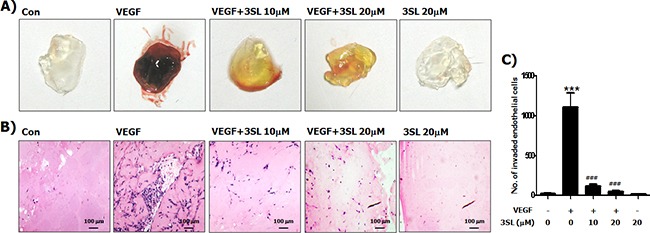
Inhibitory effect of sialyllactose on VEGF-induced *in vivo* neovascularization Matrigels were mixed with the indicated concentrations of sialyllactose in the presence of VEGF (50 ng/mL). The mixed matrigels were inoculated into the abdomen of mice. After a week, the matrigels were separated from euthanized mice. **(A)** Representative photographs and **(B)** histological images are shown. **(C)** Endothelial cells in paraffin section of Matrigel plug were counted. *** *p*<0.001 compared to the control (1^st^ lane), ### *p*<0.001 compared to the positive control (2^nd^ lane).

**Figure 6:**
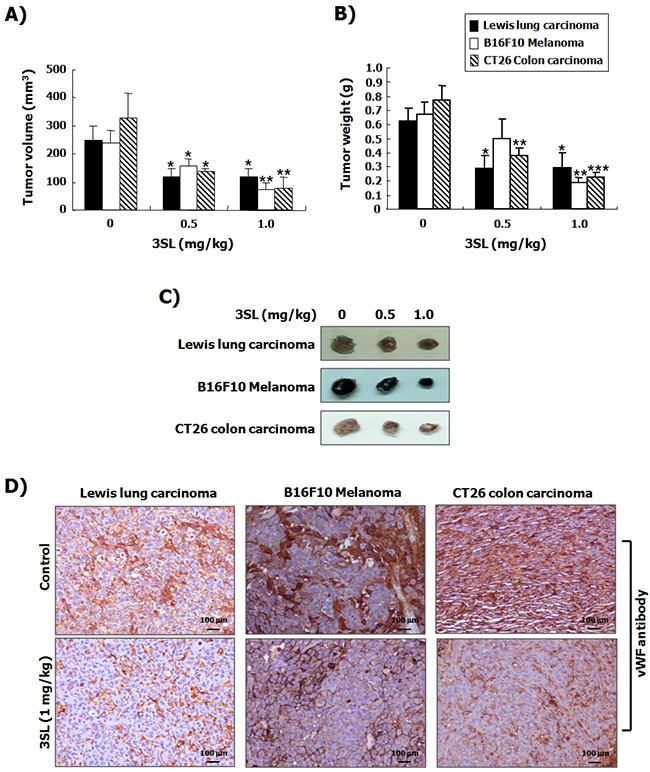
Suppression of tumor growth by sialyllactose in tumor-bearing mice Lewis lung carcinoma cells (5 × 10^5^ cells/100 μL in PBS), B16F10 melanoma cells (5 × 10^5^ cells/100 μL in PBS), and CT26 colon carcinoma cells (5 × 10^5^ cells /100 μL in PBS) were subcutaneously inoculated into the back of mice. Three days after inoculation, the indicated dosages of sialyllactose were intraperitoneally injected into mice daily. Tumor volume **(A)** and weight **(B)** were measured at the end of the experiment. The results are presented as the mean ± SD. * *p*<0.05, ** *p*<0.01 and *** *p*<0.001 compared to the control (1^st^ lane). **(C)** Representative images of each tumor treated with the indicated dose of sialyllactose are shown. **(D)** Tumor tissues were immunostained with the vWF antibody to detect newly formed vessels on tumor tissues in mice inoculated with cancer cells.

To evaluate the *in vivo* effectiveness of sialyllactose on angiogenesis, a Matrigel plug assay was performed. The results showed that VEGF-stimulated vascularization was markedly suppressed by 3SL treatment (Figure 5). In addition, the effect of 3SL on the growth of the allograft tumor was also examined. The data demonstrates that 3SL remarkably reduced the growth of three different tumors, specifically Lewis lung carcinoma, B16F10 melanoma, and CT26 colon cancer, at doses of 0.5 and 1.0 mg/kg, as measured by the volume and weight of tumors (Figure 6A-6C). 3SL also inhibited angiogenesis on allograft tumor tissues (Figure 6D). To exclude the possibility that growth inhibition could result from a direct cytotoxic effect, the *in vitro* cytotoxic effect of 3SL was evaluated. The results demonstrated that 3SL did not have any significant cytotoxic effect on the cells used for allograft (data not shown).

## DISCUSSION

Angiogenesis, the sprouting of new vessels from existing ones, is one of the hallmarks of cancer [14]. This essential step supports tumor progression by supplying nutrients and oxygen as well as enhancing tumor metastasis by serving as a gateway for entering the vascular system [15, 16]. VEGF is a primary angiogenic factor in tumor angiogenesis that drives the growth of solid tumors [17]. VEGF secreted from the tumor and stromal cells in the tumor microenvironment increases the growth and migration of vascular endothelial cells as well as the permeability of blood vessels [18]. Although several members of the VEGF family have been identified, VEGF-A, which is produced by most of cancer cells and upregulated by hypoxia, plays dominant roles in tumor angiogenesis [15, 19, 20]. In addition, of the three different VEGF receptors, VEGFR-2 is the main responder to VEGF-A in *in vivo* tumor angiogenesis [15].

Binding of VEGF-A to the ExD of VEGFR-2 and homo-dimerization of the receptors can activate subsequent signaling pathways via phosphorylation of the intracellular tyrosine kinase domain of VEGFR-2 [18, 20]. Here, we demonstrated that sialyllactose inhibits VEGFR-2 phosphorylation and VEGF/VEGFR-2-mediated intracellular signaling pathways, including phosphoinositide 3-kinase (PI3K)/Akt, ERK, and p38 in VEGF-stimulated endothelial cells (Figures 1 and 2). In addition, 3SL effectively suppressed VEGF-induced proliferation, tube formation, and migration of vascular endothelial cells (Figures 2 and 3). These results clearly demonstrate that sialyllactose plays an important role in anti-angiogenesis through suppression of VEGF-activated vascular endothelial cells. Moreover, 3SL markedly reduced the formation of VEGF-stimulated new vascular vessels and the growth of allograft tumors in *in vivo* systems (Figures 5 and 6). VEGF can also enhance the growth of some tumor cells as well as the formation of new vessels in a paracrine or autocrine manner [21]. Therefore, we confirmed whether 3SL also has an effect on tumor growth. The growth of tumor cells, including Lewis lung carcinoma, B16F10 melanoma, and CT26 colon cancer, which were used for the allograft study, were not affected by treatment of 3SL (data not shown). Based on these results, we suggest that the inhibitory effect of 3SL on the growth of allograft tumor cells is mainly due to its anti-angiogenic function.

Inhibition of VEGFR-2 activation can be achieved through diverse molecular mechanisms, such as through an inhibitor or decoy interacting with VEGF, the VEGF binding site of VEGFR-2, or the intracellular tyrosine kinase domain [18, 20–22]. In (Figure 4), we demonstrate that 3SL directly bound to the ExD of VEGFR-2, particularly the third IgG-like domain, a VEGF binding site. To investigate the structural aspects of the VEGFR-2 and 3SL interaction, we used a known third IgG-like domain (amino acids 219-330) structure of human VEGFR-2 (Figure 4D). The third IgG-like domain of VEGFR-2 is composed of eight-stranded β-sheets with two short α-helices (Figure 4C). 3SL interacts strongly with the negatively charged Asp257 and the two polar amino acids Asn259 and Ser290 of VEGFR-2 through the ionic and hydrogen bonds on the concave surface (Figure 4F).

In agreement with a previous study [13], HMOs harboring no sialic acid residue did not affect VEGF-induced phosphorylation of VEGFR-2. However, although sialyllactose is structurally similar to sialyl *N*-actetyllactosamine, only sialyllactose could suppress the activation of VEGF-mediated VEGFR-2 (Figure 1). Notably, the addition of an acetamide group to the second carbon position of glucose on sialyllactose hindered the inhibitory effect of sialyllactose on VEGFR-2 phosphorylation induced by VEGF. Similar to 3SL, sialyl *N*-actetyllactosamine interacts with the third IgG-like domain of VEGFR-2 on the concave surface (Figure 4E and 4G). Both sialyl *N*-actetyllactosamine and 3SL molecules bound to the binding pockets surrounded by β3, β4, β5, and β6 in the third IgG-like domain of VEGFR-2. However, the sialyl *N*-actetyllactosamine interacted with Asn259 and Asp257 polar residues in the pocket as well as with Asn274 at the flexible loop between α1 and β5. In this study, the interaction of sialyl *N*-actetyllactosamine with the VEGFR-2 was weaker than that with 3SL. Therefore, we postulated that the sialic acid residue and hydroxyl group on the second carbon of glucose may be essential for inhibiting VEGFR-2 activity. As shown in (Figure 4H and 4I), a Biacore analysis showed that 3SL clearly interacted with the purified second and third IgG-like domains of VEGFR-2, which are well-known VEGF binding sites. However, 3SNL did not. These results suggest that sialyllactose prevents VEGF from binding to VEGFR-2, resulting in the inhibition of VEGF-mediated VEGFR-2 activation and its downstream signaling pathways for inducing anti-angiogenesis.

Several VEGF inhibitors, such as neutralizing VEGF-A antibodies bevacizumab and ranibuzumab, VEGF trapper aflibercept, neutralizing VEGFR-2 antibody ramucirumab, and broad range VEGFR-2 tyrosine kinase inhibitors sunitinib, sorafenid, and pazopanib, are in clinical use or development [15]. However, angiogenesis inhibitors are associated with various side effects, such as hypertension, abnormal bone resorption, and renal toxicity [23–25]. These adverse effects are typical consequences of the suppression of important cellular signaling pathways that regulate and maintain the function of normal tissues [25]. Therefore, the development of selective and safe inhibitors against activation of the VEGF/VEGFR-2 axis is needed. As shown in (Figure 1), as seen with other HMOs, sialyllactose has very low toxicity. However, the selectivity of sialyllactose on VEGFR-2 and its *in vivo* toxicity should be investigated further.

In conclusion, sialyllactose has an anti-angiogenic property that suppresses the proliferation, tube formation, and migration of vascular endothelial cells through inhibition of VEGF-induced VEGFR-2 activation and consequent signaling pathways via direct interaction with VEGFR-2.

## MATERIALS AND METHODS

### Materials

The oligosaccharides of human milk, such as lactose, lacto *N*-biose, *N*-acetyllactosamine, 3′-sialyllactosamine, 6′-sialyllactosamine, 3′-sialyllactose (3SL), and 6′-sialyllactose (6SL), were purchased from Carbosynth Ltd. (Berkshire, UK). Recombinant human vascular endothelial growth factor (VEGF) was supplied by R&D systems Inc. (Minneapolis, MN, USA). Antibodies against the phosphorylated form or total form of VEGF receptor-2 (VEGFR-2), extracellular signal-regulated kinase (ERK), Akt, and p38 were purchased from Cell Signaling Technology (Danvers, MA, USA). The antibody against von Willebrand Factor (vWF) was purchased from Dako (CA, USA). Antibodies against glyceraldehyde 3-phosphate dehydrogenase (GAPDH) and hoseradish peroxide (HRP)-conjugated secondary antibodies were supplied by Santa Cruz Biotechnology (Santa Cruz, CA, USA).

### Cell culture

HUVECs were purchased from Cambrex Inc. (Walkersville, MD, USA) and cultured in sterile endothelial growth medium-2 (EGM-2, Cambrex Inc.). HUVECs at passage number 5 to 8 were used in experiments. Murine lewis lung carcinoma (LLC) cells were purchased from American Type Culture Collection (Manassas, VA, USA). Mouse melanoma (B16F10) and colon carcinoma (CT26) cells were provided by the Korean Cell Line Bank (Seoul, Korea). The cells were cultured with Dulbecco Modified Eagle Medium4 (ThermoFisher Scientific) supplemented with 10% heat-inactivated fetal bovine serum (Sigma-Aldrich, St. Louis, MO, USA), 100 unit/mL of penicillin, and 100 μg/mL of streptomycin. All cell lines were maintained at 37 °C in a humidified 5 % CO_2_ cell culture incubator.

### Cell viability

The cytotoxic effects of HMOs were examined using the methylthiazolyldiphenyl-tetrazolium bromide assay (MTT, Sigma-Aldrich). In brief, HUVECs were cultured in 24-well plates including endothelial basic medium-2 (EBM-2, Cambrex Inc.) containing 1 % FBS with the indicated concentration of oligosaccharides in the presence or absence of VEGF (50 ng/mL; R&D System Inc.) for 72 h. After removal of the culture medium, 300 μL of 1× MTT solution (0.5 mg/mL) was added to each well. After incubation for 4 h at 37°C in a CO_2_ incubator, the supernatant was removed, and the formed formazan crystals in viable cells were dissolved in 300 μL of ethanol:DMSO (v/v, 1:1). To detect cytotoxicity, the absorbance in each well was measured at 540 nm using a SpectraMax M2 reader (Molecular Devices, Sunnyvale, CA, USA). The percentage of living cells in treated cultures was calculated relative to that in untreated cultures.

### Western blot analysis

Total protein from HUVECs treated with the indicated concentration of oligosaccharides in the presence or absence of VEGF was extracted using 1% NP-40 lysis buffer [150mM NaCl, 10mM HEPES (pH 7.45), 1% NP-40, 5 mM NaPyrophosphate, 5mM NaF, 2mM Na3VO4] containing protease inhibitor cocktail tablet (Roche, Mannheim, Germany). Equal amounts (30 μg) of proteins were used for western blot analysis. To detect target proteins, membranes were incubated with a 1:1000 dilution of primary antibodies against each protein, and reacted with the corresponding HRP-conjugated secondary antibodies. Bands were detected with Pierce ECL plus (ThermoFischer Scientific, Waltham, MA, USA) using ImageQuant LAS4000 (GE healthcare, Pittsburgh, PA, USA).

### Tube forming assay

The capillary-like network formation of HUVECs was investigated with Matrigel-coated 24-well culture plates, as previously described [19]. Matrigel (13.9 mg/mL; BD Bioscience, San Jose, CA, USA) was thawed at 4 °C and mixed with the same volume of EBM-2 medium. Seventy microliters of the mixture (6.95 mg/mL of Matrigel) was added to each well of the 24-well culture plates and incubated at 37 °C for 1 h for polymerization. The HUVECs were suspended in EBM-2 medium containing 1 % FBS (1×10^4^ cells/well), and were then added to the Matrigel-coated wells at the indicated concentrations of 3SL in the presence or absence of VEGF. The plates were incubated at 37 °C in 5 % CO^2^ atmosphere. After 15 h incubation, the capillary-like tube formation in each well of the culture plates was photographed with a Nikon Eclipse TS100 microscope (Nikon, Tokyo, Japan).

### Migration assay

The migration assay of HUVECs was performed using 24-well chambers containing polycarbonate filter inserts (Corning Inc., Corning, NY, USA), as previously described [19] with some modification. The HUVECs were suspended in EBM-2 medium containing 1 % FBS (1×10^3^ cells/ 200 μL), and were added to the upper compartment of the chamber with the indicated concentrations of 3SL. EBM-2 medium containing 1 % FBS with or without VEGF was added to the lower chamber. The chambers were incubated at 37 °C and in 5 % CO_2_ atmosphere. After 24 h, the HUVECs on the upper side of the filter were removed using cotton swabs. The filters were fixed, stained, and mounted on microscope slides. The migrated HUVECs to the lower side of the filter were counted. The results were calculated as the average of migrated cell numbers from three different filters.

### Wound healing assay

To check migration of unidirectional HUVEC, wound healing assay was performed. The HUVECs (5 × 10^5^ cells/well) were seeded in 6-well plates, and cultured in EGM-2 medium. When the cells confluently grew, HUVEC monolayers were scratched with a 10–200 μL pipette tip to make a straight line on the HUVEC monolayer. The cells were treated with or without VEGF in the presence or absence of 3SL, and incubated for 12 h at 37 °C and 5 % CO_2_ atmosphere. Images of the cells were obtained with a Nikon Eclipse TS100 microscope (Nikon, Tokyo, Japan) at 0 h and 12 h.

### Immunofluorescence microscopy

Immunofluorescence staining was performed as previously described [19]. Briefly, HUVECs were seeded on 12 mm π-sterilized coverslips in 24-well tissue culture plates, and treated with the indicated concentration of 3SL in the presence or absence of VEGF. After 24 h, the cells were fixed in 3.7 % formalin, and permeabilized with 0.5 % Triton X-100 in PBS. After washing three times with PBS, the cells were blocked with PBS containing 1 % bovine serum albumin at room temperature with gentle shaking. Afterwards, a solution (1:50 dilution) of anti-paxillin antibody was added to the cells and followed by incubation at 4 °C overnight. The samples were further incubated with 2 μg/ml of Alexa Fluor 488-conjugated goat anti-rabbit IgG (Invitrogen) for 1 h at room temperature. After washing with PBS, the samples were incubated with PBS including 1 unit of Texas Red®-X phalloidin (Invitrogen) for 30 min at room temperature for actin staining. After washing again with PBS, the samples were analyzed under a fluorescence microscope.

### Immunoprecipitation and high performance thin layer chromatography (HPTLC)

To investigate whether 3SL interacts with VEGFR-2, the purified and identified ExD of VEGFR-2 proteins were used. The VEGFR-2 ExD protein (1 μg) was bound with 3SL (30 μM) in 300 μL of IP buffer at 4°C overnight. The anti-VGFER-2 ExD antibody (2 μg) was added to 3SL and VEGFR-2 ExD protein mixtures and incubated at 4°C overnight with shaking. The sample was incubated with 20 μL of the protein G PLUS-agarose beads (ThermoFisher Scientific) for 4 h at 4°C. 3SL interacting with VEGFR-2 ExD was released from the beads through the addition of methanol, and applied to an HPTLC plate with chloroform–methanol–0.2% CaCl_2_ (55:45:10). After separation, 3SL was visualized by spraying the plates with a resorcinol-hydrochloric acid reagent.

### Simulation of protein-carbohydrate binding

Both the 3SL and sialyl *N*-actetyllactosamine interact with ExD of VEGFR-2 (PDB ID: 3S35) as determined by a protein-small molecule docking method. The three-dimensional structure of VEGFR-2 IG3 domain was identified from the RCSB Protein Data Bank and submitted to SwissDock for protein-ligand docking prediction with 3SL and sialyl *N*-actetyllactosamine structure information.

### Expression and purification of VEGFR-2 IG3

The vector expressing the second and third IgG-like domain of VEGFR-2 (VEGFR-2 IG3) was constructed according to a previous study [26]. The vector was transformed into overexpression competent cells, *Escherichia coli* BL21(DE3). Each colony was inoculated in 5 mL of Luria Bertani (LB) medium enriched with 10 μg/mL kanamycin at 37°C overnight. The cells were then incubated in 2 L of LB containing 10 μg/mL antibiotics at 37°C until the OD_600_ reached 0.5-0.6. Next, the expression of VEGFR-2 IG3 was induced by 0.5 mM isopropyl-thio-β-d-galactopyranoside (IPTG) at 20°C overnight, and the bacterial cells were then harvested by centrifugation at 3,660 × g for 25 min at 4°C. The cell pellets were resuspended in lysis buffer [50 mM glycine (pH 10.5)] containing a protease inhibitor cocktail (Roche Diagnostics), and then sonicated on ice to disrupt the cells using a sonicator (Branson Sonifier 450 sonicator; Danbury, USA). The cell suspensions were centrifuged at 20,170 × g for 45 min to separate the supernatant and pellet. Lysis was repeated four times and the final supernatant was concentrated using Vivaspin 20 and centrifuged at 1,320 × g. Finally, the concentrated fractions of VEGFR-2 IG3 were subsequently purified by gel filtration chromatography using a Superdex 200 10/300 GL fast protein liquid chromatography (FPLC) column (GE Healthcare, Sweden) equilibrated in 50 mM Tris-HCl (pH 8.0) and 200 mM NaCl. Concentrated VEGFR-2 IG3 was analyzed by 15% sodium dodecyl sulfate polyacrylamide gel electrophoresis (SDS-PAGE).

### Surface plasmon resonance (SPR) biosensor analysis

Measurements of the apparent dissociation constants (K_D_) between VEGFR-2 IG3 and chemical compounds were carried out using a Biacore T100 biosensor (GE Healthcare Biosciences, Sweden). The purified VEGFR-2 IG3 domain was covalently bound to the Series S sensor chip CM5 (carboxylated dextran matrix) using an amine-coupling method, as suggested by the manufacturer. The 150 μL of VEGFR-2 (50 μg/mL) in 10 mM sodium acetate pH5.0 was coupled via injection for 15 min at 10 μL/min, followed by injection of 1 M ethanolamine to deactivate the residual amines. For kinetic measurements at 25°C, chemical compounds with concentrations ranging from 120 to 7.5 μM were prepared by dilution in HBS-EP^+^ buffer (10 mM of HEPES, 150 mM of NaCl, 3 mM of EDTA, and 0.005% v/v surfactant P20) at a pH of 7.4. The immobilized ligand was regenerated by injecting 10 μL of 50 mM NaOH at a rate 10 μL/min during the cycles.

### Animals

Male C57BL/6 and BALB/c mice (6-7 weeks old, weight 20-22 g), inbred in a specific pathogen-free (SPF) facility, were purchased from Orient Bio, Co. (Seongnam, Korea). The mice were bred separately and had free access to water and a standard diet with a 12 h light: 12 h dark cycle. All experimental procedures were examined and approved by the Animal Research Ethics Committee at the Pusan University of Korea.

### Matrigel plug assay

The Matrigel plug assay was performed as previously described [19]. Briefly, C57BL/6 mice were subcutaneously injected with 500 μL of a BD Matrigel Matrix and heparin (50 unit/mL; BD Bioscience) mixture with the indicated concentration of 3SL in presence or absence of VEGF (100 ng/mL). Five male mice were assigned to each group. After 7 days, the matrigel plugs were removed from the euthanized mice and fixed with 3.7 % formalin in PBS. The plugs were embedded in paraffin, and cut into 4 μm sections. The sections were stained with hematoxylin and eosin (H&E) solutions for microscopic observation.

### Tumor allograft

Each cell line, specifically LLC, B16F10, and CT26, was suspended in PBS (5 × 10^5^ cells/100 μL) and subcutaneously inoculated in the dorsa of 6-7 week-old C57BL/6 (for LLC and B16F10) and BALB/c mice (for CT26). Six male mice were assigned to each group. Three days after the injection of tumor cells, 3SL (0.5 or 1 mg/kg in PBS) or PBS was intraperitoneally (i.p.) injected once per day for 12 days. The tumors were excised and weighed 15 days after inoculation of tumor cells. Tumor volumes were measured with a pair of calipers, and were calculated according to the formula [(length × width)^2^/2].

### Immunohistochemistry

Tumor specimens were immediately removed from the euthanized tumor-bearing mice, fixed with 3.7 % formalin in PBS, and then embedded in paraffin for immunohistochemical analysis. The paraffin sections were immunostained with an antibody against the endothelial cell marker vWF, visualized using Dako EnVision kit (Dako, CA, USA), and counterstained with hematoxylin.

### Statistical analysis

The differences between the mean values of experiment groups were determined by one-way analysis of variance (one-way ANOVA) with a Turkey's post-hoc test, using GraphPad Prism (Graphpad Software, San Diego, CA, USA). The minimum significance level was set at a *p* value of 0.05. All experiments were independently performed at least 3 times.

## SUPPLEMENTARY MATERIALS FIGURES AND TABLE


