# Supplementary material for: Sialyllactose suppresses angiogenesis by inhibiting VEGFR-2 activation, and tumor progression
Source: Oncotarget. 2017 Mar 14;8(35):58152–62. doi: 10.18632/oncotarget.16192 (PMC5601640; doi:10.18632/oncotarget.16192)
Supplement: Supplementary file 1 [file oncotarget-08-58152-s001.pdf]

Sialyllactose suppresses angiogenesis by inhibiting VEGFR-2 activation, and tumor progression

SUPPLEMENTARY MATERIALS

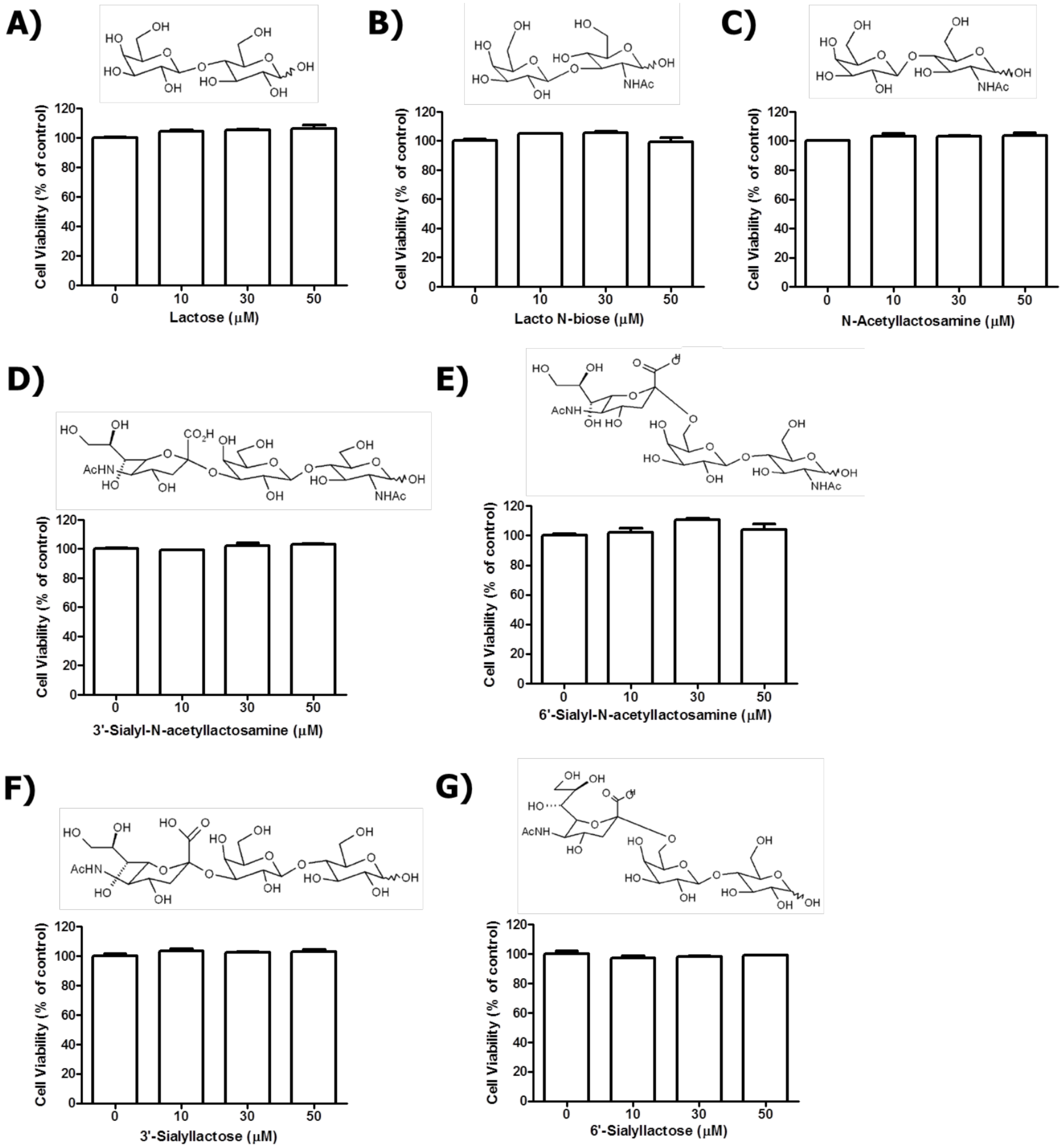

Supplementary Figure 1: Effect of milk sialic oligosaccharides on the growth of HUVECs. **(A-G)** The structures of representative milk sialic oligosaccharides were demonstrated. The HUVECs were treated with indicated concentrations of each oligosaccharide for 24 h. The viabilities of HUVECs were evaluated by MTT assay.

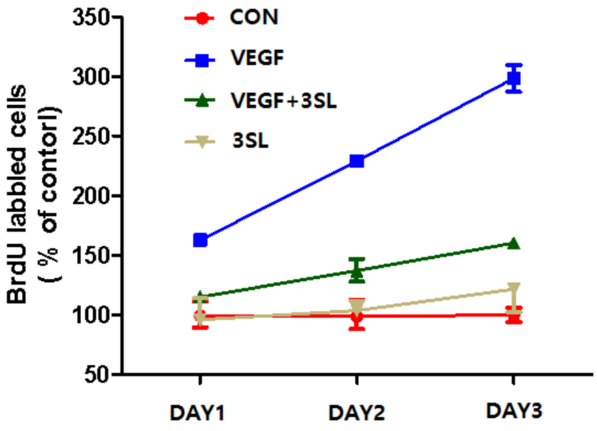

Supplementary Figure 2: Effect of SL on the growth of HUVECs stimulated by VEGF. (A-G) The HUVECs were treated with or without SL (30 μM) in the presence or absence of VEGF (50 ng/ml) for the indicated times. The viabilities of HUVECs were evaluated by 5-bromo-2-deoxyuridine (BrdU) cell proliferation assay (BioVision, CA, USA).

Supplementary Table 1: Effect of SL on the liver and kidney function in mice

**LLC-inoculated**			
**Dose (mg/kg)**	**0**	**0.5**	**1.0**
AST (Unit/L)	430.8±140.7	419.6±85.6	406.6±82.6
ALT (Unit/L)	58.3±8.7	46.6±10.8	51.6±4.08
BUN (mg/dL)	25.1±9.7	21.2±1.6	21.4±3.1
Creatinine (mg/dL)	<0.2	<0.2	<0.2
**B16-F10-inoculated**			
AST (Unit/L)	450.8±71.2	438.3±44.4	420.0±38.0
ALT (Unit/L)	47.5±10.3	44.1±6.6	45.0±8.9
BUN (mg/dL)	25.1±4.8	21.8±1.8	21.9±2.1
Creatinine (mg/dL)	<0.2	<0.2	<0.2
**CT26-inoculated**			
AST (Unit/L)	390.0±58.4	378.3±97.5	374.1±59.2
ALT (Unit/L)	43.3±12.9	40.8±10.2	40.0±7.7
BUN (mg/dL)	24.8±9.8	20.0±2.2	21.3±1.4
Creatinine (mg/dL)	<0.2	<0.2	<0.2

At the end of the *in vivo* allograft experiment, blood of each mouse was collected from the retro-orbital plexus, and then the sera from blood were prepared. To investigate the toxicity of SL on liver and kidney of mice, biochemical analysis on the aspartate aminotransferase (AST), alanine aminotransferase (ALT), creatinine, and blood urea nitrogen (BUN) were performed by commercial service by Green Cross Co. (Yongin, Korea).
